# Author Correction: Improved prediction of protein-protein interactions using AlphaFold2

**DOI:** 10.1038/s41467-022-29480-5

**Published:** 2022-03-24

**Authors:** Patrick Bryant, Gabriele Pozzati, Arne Elofsson

**Affiliations:** 1 Science for Life Laboratory, 172 21 Solna, Sweden; 2 Department of Biochemistry and Biophysics, Stockholm University, 106 91 Stockholm, Sweden

**Keywords:** Structural biology, Protein structure predictions, Proteome informatics

Correction to: *Nature Communications* 10.1038/s41467-022-28865-w, published online 10 March 2022.

The original version of this article contained an error in the Methods section, which incorrectly read:

‘This score is created by fitting a sigmoidal curve (Fig. 2c) using “curve_fit” from SciPy v.1.4.1^56^, to the DockQ scores using the average interface plDDT multiplied with the logarithm of the number of interface contacts, with the following sigmoidal equation:

7 $${{{\rm{pDockQ}}}}=\frac{L}{1+e^{-k(x-x_0)}}+{{{\rm{b}}}}$$

where

*x* = average interface plDDT⋅log(number of interface contacts) (8) and we obtain *L* = 0.958, *x*_0_ = 160.11, *k* = 1 and *b* = 0.001.’

The correct version states ‘*L* = 0.724, *x*_0_ = 152.611, *k* = 0.052 and *b* = 0.018’ in place of ‘*L* = 0.958, *x*_0_ = 160.11, *k* = 1 and *b* = 0.001.’

This has been corrected in both the PDF and HTML versions of the article.

