# Supplementary material for: Improved prediction of protein-protein interactions using AlphaFold2
Source: Nat Commun. 2022 Mar 10;13:1265. doi: 10.1038/s41467-022-28865-w (PMC8913741; doi:10.1038/s41467-022-28865-w)
Supplement: Supplementary file 1 — Supplementary Information [file 41467_2022_28865_MOESM1_ESM.pdf]

Supplementary Information for

Improved prediction of protein-protein interactions using AlphaFold2

Patrick Bryant[1,2,*], Gabriele Pozzati[1,2] and Arne Elofsson[1,2*]

[1]Science for Life Laboratory, 172 21 Solna, Sweden
[2]Department of Biochemistry and Biophysics, Stockholm University, 106 91 Stockholm, Sweden

Supplementary Tables

Supplementary Table 1.
Pearson correlation coefficients of DockQ sets from different modelling setups

**Model**	model 1						model 1 ptm					
**MSA**	paired		AF2		AF2+paired		paired		AF2		AF2+paired	
**Strategy**	ens 8	rec 10	ens 8	rec 10	ens 8	rec 10	ens 8	rec 10	ens 8	rec 10	ens 8	rec 10
Setup ID	1	2	3	4	5	6	7	8	9	10	11	12
1	0,00	**0,96**	0,51	0,53	0,78	0,72	**0,98**	**0,93**	0,57	0,53	0,81	0,75
2	**0,96**	0,00	0,53	0,54	0,78	0,72	**0,95**	**0,90**	0,56	0,52	0,81	0,75
3	0,51	0,53	0,00	**0,89**	0,72	0,66	0,51	0,54	**0,81**	**0,78**	0,64	0,63
4	0,53	0,54	**0,89**	0,00	0,65	0,62	0,53	0,56	**0,80**	**0,75**	0,68	0,64
5	0,78	0,78	0,72	0,65	0,00	**0,89**	0,79	0,74	0,65	0,64	**0,89**	**0,86**
6	0,72	0,72	0,66	0,62	**0,89**	0,00	0,72	0,67	0,60	0,62	**0,83**	**0,83**
7	**0,98**	**0,95**	0,51	0,53	0,79	0,72	0,00	**0,94**	0,56	0,51	0,81	0,75
8	**0,93**	**0,90**	0,54	0,56	0,74	0,67	**0,94**	0,00	0,59	0,51	0,77	0,74
9	0,57	0,56	**0,81**	**0,80**	0,65	0,60	0,56	0,59	0,00	**0,90**	0,71	0,68
10	0,53	0,52	**0,78**	**0,75**	0,64	0,62	0,51	0,51	**0,90**	0,00	0,67	0,66
11	0,81	0,81	0,64	0,68	**0,89**	**0,83**	0,81	0,77	0,71	0,67	0,00	**0,93**
12	0,75	0,75	0,63	0,64	**0,86**	**0,83**	0,75	0,74	0,68	0,66	**0,93**	0,00

Pearson correlation coefficients of DockQ sets from different modelling setups on the development set (n=216). Bold labels highlight modelling setup variables, while bold values indicate correlation values between the same input MSAs.

**Supplementary Table 2.** Quality metrics for test set AF2 ranked models					
TPR	FPR	PPV	Fraction Correct	Fraction Incorrect	pDockQ
0.370	0.007	0.981	0.228	0.004	0.674
0.416	0.016	0.963	0.257	0.010	0.655
0.504	0.025	0.953	0.311	0.015	0.629
0.525	0.034	0.940	0.324	0.021	0.622
0.578	0.042	0.932	0.357	0.026	0.596
0.624	0.051	0.924	0.385	0.032	0.577
0.656	0.060	0.916	0.405	0.037	0.558
0.691	0.069	0.910	0.427	0.042	0.535
0.718	0.079	0.900	0.443	0.049	0.516
0.757	0.092	0.892	0.467	0.057	0.490
0.779	0.101	0.886	0.481	0.062	0.471
0.814	0.113	0.878	0.502	0.070	0.441
0.835	0.123	0.871	0.515	0.076	0.421
0.848	0.138	0.860	0.523	0.085	0.396
0.860	0.146	0.855	0.531	0.090	0.381
0.871	0.162	0.843	0.537	0.100	0.362
0.884	0.176	0.834	0.546	0.109	0.344
0.898	0.194	0.822	0.554	0.120	0.326
0.906	0.210	0.812	0.559	0.130	0.315
0.916	0.226	0.802	0.565	0.139	0.2
0.930	0.254	0.785	0.574	0.157	0.269
0.938	0.268	0.778	0.579	0.165	0.249
0.946	0.300	0.759	0.584	0.185	0.223
0.954	0.335	0.740	0.589	0.207	0.201
0.961	0.354	0.730	0.593	0.219	0.181
0.968	0.388	0.714	0.598	0.239	0.160
0.975	0.406	0.706	0.602	0.250	0.145
0.980	0.448	0.686	0.605	0.276	0.126
0.987	0.492	0.667	0.609	0.304	0.106
0.993	0.570	0.636	0.613	0.352	0.074
0.999	0.788	0.559	0.616	0.487	0.030

Presented metrics are derived from models of the test set (n=1,481), obtained by selecting the highest pDockQ model from 5 AF2 runs with AF+paired MSAs and m-10-1 strategy (see Table 1 in the main text). False Positive Rates (FPR) and True Positive Rates (TPR) are calculated using pDockQ thresholds to classify selected models as correct or incorrect. Additionally, the fraction of correct and incorrect models and the Positive Predictive Value (PPV) are reported. The correct and incorrect fractions are obtained by respectively multiplying the TPR and FPR with the success rate (SR=0.617), while PPV is obtained by dividing the TPR with TPR+FPR.

**Supplementary Table 3.** Results on CASP14 and new heterodimers			
PDB	Chain 1	Chain 2	Top-ranked DockQ score
CASP 14 heterodimers			
7M5F	A	C	0.85
6XOD	A	B	0.94
6VN1	A	H	0.01
6VN1	H	L	0.45
6R17	A	C	0.54
6TMM*	A	B	0.03
6TMM*	A	D	0.04
Heterodimers without templates			
7EIV	A	C	0.76
7EL1	A	E	0.01
7K01	1	6	0.36
7LDG	A	B	0.45
7LF7	A	M	0.02
7LF7	B	M	0.02
7MEZ	A	B	0.53
7NJ0	A	C	0.04

The two interactions involving 6TMM [https://files.rcsb.org/download/6TMM.cif]. are different possible configurations of the same two chains.

Supplementary Figures

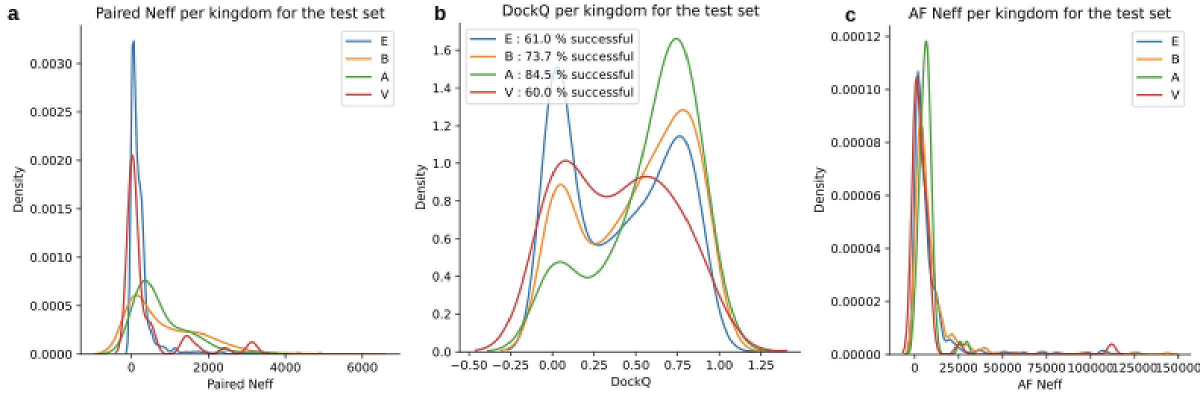

Supplementary Figure 1. Distribution of different test set (n=1,481) features divided by kingdom. In the figure different features A) Paired Neff, B) DockQ scores, and C) AF Neff are plotted according to complex provenience from: E=Eukarya, B=Bacteria, A=Archaea and V=Virus.

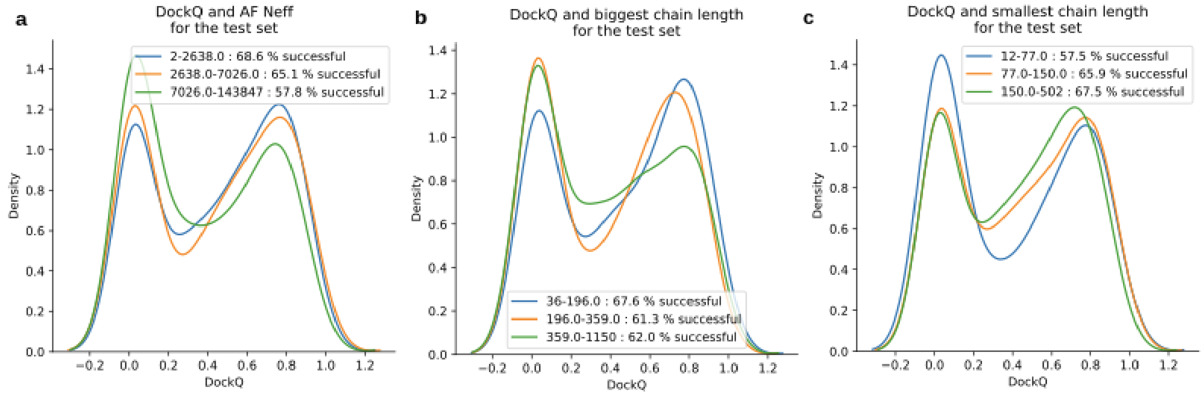

Supplementary Figure 2. DockQ distributions for test dataset (n=1,481) tertiles. Tertiles are derived from A) AF Neff B) biggest chain lengths and C) smallest chain length. The separation between the tertiles is low for all features displaying similar success rates.

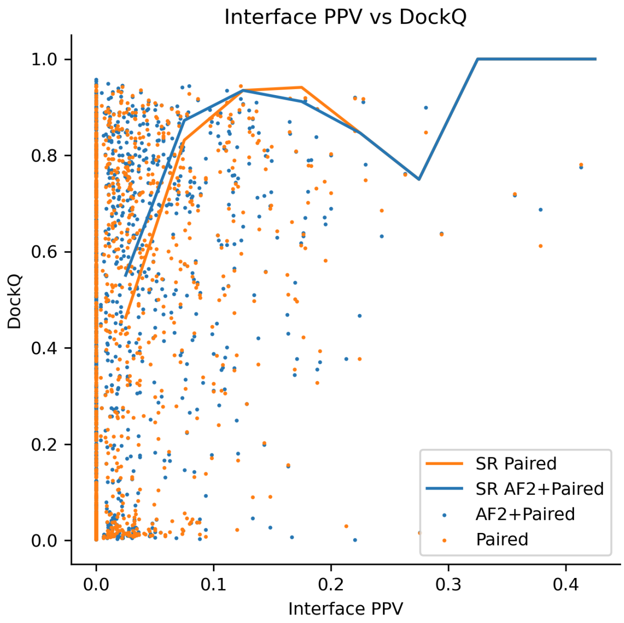

Supplementary Figure 3. Interface PPV in paired MSAs vs DockQ scores and success rates (SR) on the test set (n=1,481). The SR increases with interface PPV for both paired and AF2+paired modelings, suggesting a strong influence of the MSAs on the outcome.

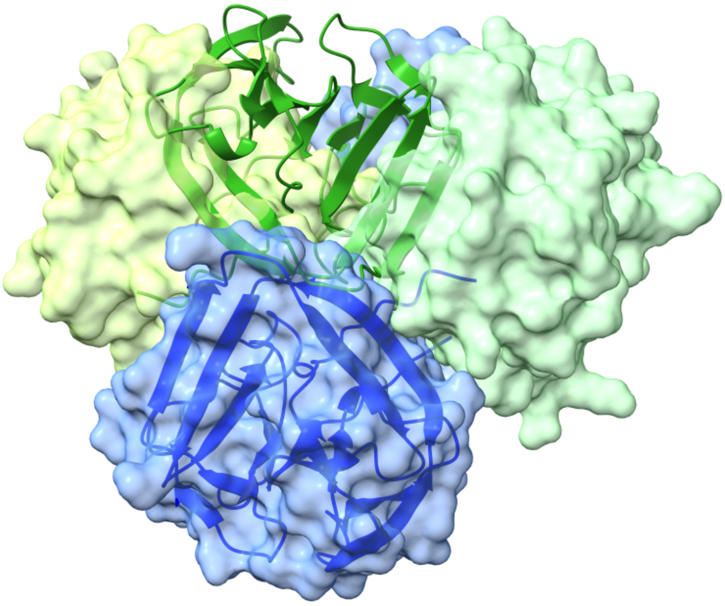

Supplementary Figure 4. Model of CASP14 heterodimer from PDB 6TMM [https://files.rcsb.org/download/6TMM.cif]. The model obtained using AF2 default and paired MSA (ribbons) is superposed to the native heterocomplex (surfaces). The smaller model chain (green ribbon) is positioned halfway between the two alternative binding sites formed by blue and green surfaces.

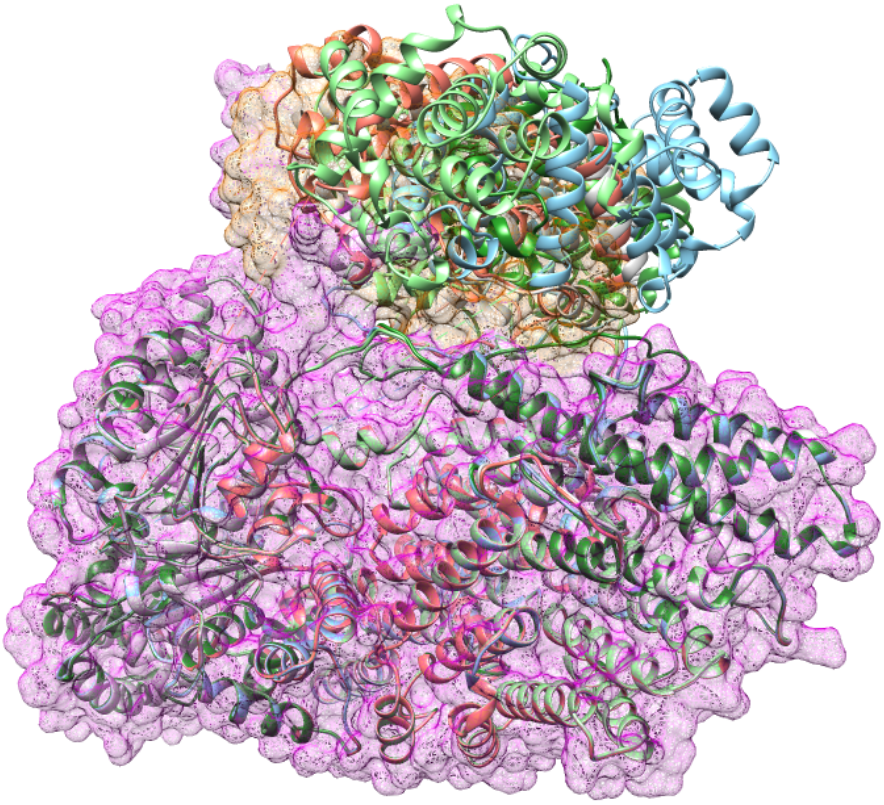

Supplementary Figure 5. Model of heterodimer from PDB 7NJ0 [https://files.rcsb.org/download/7NJ0.pdb]. The native structure is represented as a mesh surface (orange and magenta). All predictions (ribbons) get the location of the chains correct, but the interface and orientations are slightly wrong, resulting in DockQ scores close to 0.
